# Supplementary figures and images for: Protein flexibility is required for vesicle tethering at the Golgi
Source: eLife. 2015 Dec 14;4:e12790. doi: 10.7554/eLife.12790 (PMC4721967; doi:10.7554/eLife.12790)

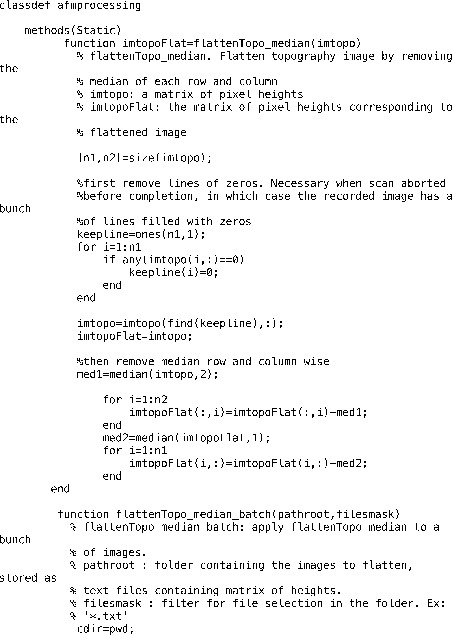

Supplement: Source code 1. — DOI: http://dx.doi.org/10.7554/eLife.12790.015 [file elife-12790-code1.jpg]
